# Changing face-to-face psychological care to remote mode: facilitators and obstacles in the COVID-19 pandemic *

**DOI:** 10.1590/1518-8345.6468.3900

**Published:** 2023-04-17

**Authors:** Jorge Henrique Correa dos Santos, Pamela Perina Braz Sola, Manoel Antônio dos Santos, Érika Arantes de Oliveira-Cardoso

**Affiliations:** 1 Universidade de São Paulo, Faculdade de Filosofia Ciências e Letras, Ribeirão Preto, SP, Brasil

**Keywords:** Online Therapy, Information Technology, COVID-19, Pandemic, Psychotherapy, Psychology, Terapia en Línea, Tecnología de la Información, COVID-19, Pandemia, Psicoterapia, Psicología, Terapia Online, Tecnologia da Informação, COVID-19, Pandemia, Psicoterapia, Psicologia

## Abstract

**Objective:**

to verify associations between sociodemographic variables and factors that facilitate and hinder the transition from face-to-face psychological care to remote mode in the first year of the COVID-19 pandemic.

**Method:**

this is an analytical, quantitative, cross-sectional study. After approval by the Research Ethics Committee, data collection was performed by applying an online form consisting of 55 questions. Data were analyzed using descriptive and inferential statistics techniques.

**Results:**

the intentional sampling consisted of a total of 385 Brazilian psychologists, mostly women (67.01%), young professionals with up to five years of graduation (44.16%) most of activities in the private clinic. It was found that training time between five and 10 years was associated with a greater perception of difficulties and that previous experience with remote care facilitated adaptation in the transition from one modality to another.

**Conclusion:**

considering that call center can be a powerful tool in the health scenario, it is suggested the inclusion of remote care issues in the research agenda and syllabus in the curricula of health training courses.

## Abstract (es)

**Objetivo:**

verificar asociaciones entre variables sociodemográficas y factores que facilitan y dificultan la transición de la atención psicológica presencial a la modalidad remota en el primer año de la pandemia de COVID-19.

**Método:**

se trata de un estudio analítico, cuantitativo y de corte transversal. Después de la aprobación del Comité de Ética en Investigación, la recolección de datos se realizó mediante la aplicación de un formulario en línea que consta de 55 preguntas. Los datos fueron analizados utilizando técnicas de estadística descriptiva e inferencial.

**Resultados:**

la muestra de conveniencia estuvo compuesta por 385 psicólogos brasileños, en su mayoría mujeres (67,01%), jóvenes profesionales con hasta cinco años de actuación después de la graduación (44,16%) y predominio de actividades en la clínica privada. Se encontró que el tiempo de formación entre cinco y 10 años se asoció con una mayor percepción de dificultades y que la experiencia previa con la atención a distancia facilitó la adaptación en la transición de una modalidad a otra.

**Conclusión:**

considerando que la teleasistencia puede ser una poderosa herramienta en el escenario de la salud, se sugiere que los temas de la teleasistencia sean incluidos en la agenda de investigación y los contenidos programáticos en los currículos de los cursos de formación en salud.

## Abstract (pt)

**Objetivo:**

verificar associações entre variáveis sociodemográficas e fatores facilitadores e dificultadores da transição do atendimento psicológico presencial para a modalidade remota no primeiro ano da pandemia de COVID-19.

**Método:**

trata-se de um estudo analítico, quantitativo, de corte transversal. Após aprovação do Comitê de Ética em Pesquisa, a coleta foi realizada mediante aplicação de um formulário online composto por 55 questões. Os dados foram analisados por meio de técnicas de estatística descritiva e inferencial.

**Resultados:**

a amostra de conveniência foi composta por 385 psicólogos brasileiros, majoritariamente mulheres (67,01%), jovens profissionais com até cinco anos de ofício após a graduação (44,16%) e com predomínio de atividades na clínica privada. Constatou-se que o tempo de formação entre cinco e 10 anos foi associado com uma maior percepção de dificuldades e que a experiência prévia com atendimento remoto foi facilitadora da adaptação na transição de uma modalidade à outra.

**Conclusão:**

considerando que o teleatendimento pode ser uma ferramenta potente no cenário da saúde, sugere-se a inclusão das questões do atendimento remoto na agenda de pesquisa e conteúdos programáticos das grades curriculares dos cursos de formação em saúde.

**Highlights:**

**(1)** Most professionals considered remote care more tiring.

**(2)** Less training time was a facilitating factor in the transition to online care.

**(3)** Not having previous experience with remote service was a difficult factor.

**(4)** Psychologists still expressed doubts and insecurities regarding online assistance.

**(5)** It is recommended to include technology-mediated care in the undergraduate curriculum.

## Introduction

During the Coronavirus Disease 2019 (COVID-19) pandemic, health services implemented adaptations in their functioning in order to meet the growing demand and ensure continuity of care for other diseases ^( 1- 3)^ , generating systemic impacts throughout the health care chain in general ^( 4- 6)^ , including mental health. In this scenario, telework ^( 7)^ , or telehealth ^( 8)^ constituted a possibility of preserving the continuity of health care. 

Despite its notable diffusion in the last decade, call center is still far from having wide and unrestricted acceptance among mental health professionals. One of the biggest challenges faced was the need to review and transform the pillars of an established practice, which led to the creation of an atmosphere of distrust regarding the viability of remote psychotherapy, despite studies showing its effectiveness and feasibility ^( 9- 10)^ . 

If on the one hand there was ample room for the reinvention of the therapeutic setting and the revitalization of clinical practice, on the other hand, the emergency nature of the transition to the remote environment produced sudden changes, often based on improvisation and on trial-and-error ^( 11- 12)^ . The professionals were required, in addition to adapting their technique, to be able to deal with the unforeseen events typical of technology-mediated communication, such as possible problems with network connection, choice of platform, guarantee of privacy and preservation of secrecy in an adapted environment ^( 13- 14)^ . 

Considering that these new challenges were inserted in a context of collective suffering imposed by the pandemic, professionals needed to respond to a growing demand for mental health care. A study that investigated symptoms of anxiety, depression and stress in the Brazilian population during the pandemic found that 46.4% of participants had symptoms of depression, 42.2% of stress and 39.7% of anxiety ^( 15)^ . This urgent character and demand pressure meant that the transition to non-face-to-face psychotherapy occurred without any support from supervision or prior training of professionals ^( 16)^ , which may have contributed to the difficulties mentioned. The pandemic generated stagnation in the economy, paralyzed production chains, aroused distress in families, increased psychosocial suffering ^( 17)^ and produced an unprecedented number of lost lives ^( 18- 19)^ . However, in terms of organizing work activities, the adverse scenario also created a window of opportunity to leverage substantial changes in care modes and review traditional ways of exercising professional practice, with the possibility of making gains with remote intervention. Technology-mediated care favors greater flexibility of schedules, does not require travel around the city, involves less financial investment, with the possibility of greater monetary return ^( 13)^ . 

Considering that the COVID-19 pandemic has contributed to highlighting that health crises require professionals to broaden their understanding of health, with a view to understanding the interrelationship between the threat to physical integrity and psychological suffering ^( 20)^ , it is relevant to know which were the facilitating and hindering factors found by Psychology professionals in this context. It starts from the assumption that emotional support was – and possibly will still be – fundamental for facing the health crisis, which calls for the planning of preventive and interventional actions in this scenario. The knowledge may help other health professionals who also had to adapt to the demands of remote care, without due preparation or prior training. 

Considering these assumptions, the objective of this study is to verify associations between sociodemographic variables and factors that facilitate and hinder the transition from face-to-face psychological care to remote mode in the first year of the COVID-19 pandemic.

## Method

### Study design and period

This is a quantitative, analytical, cross-sectional study. The STROBE guide (Strengthening the Reporting of Observational Studies in Epidemiology) of the Enhancing the QUAlity and Transparency Of health Research Network (EQUATOR) was used to organize the text. Data were collected during May to November, 2020.

### Casuistry

The sample, non-probability convenience sampling ^( 21)^ , consisted of 385 psychologists. The research was publicized on social networks and in WhatsApp messaging groups, with a view to recruiting potential participants. 

The inclusion criteria in the study were: to have an active record in the Regional Council of Psychology (RCP) of the participant’s reference, to perform remote clinical psychological care, to have previously carried out face-to-face psychological care. The exclusion criteria were: not having access to a quality device or connection to complete the online form.

### Instrument

For data collection, a questionnaire developed using online forms was used, which is advantageous due to its gratuity, ease of distribution, completion and verification of results ^( 22)^ . The online form was prepared on the Google Forms platform, compatible with both desktop computers and mobile devices, which can facilitate access by participants. One advantage of using this type of file is that, as it is stored on Google’s server, it can be accessed from any computer connected to the Internet, replacing printed instruments and allowing better access and organization of data. In addition, form answers are automatically grouped into graphs, which can be retrieved in different file formats. To prevent the form from being modified, deleted or shared by research participants, its editing was restricted to the researcher who created the online form. 

The form was prepared by the first author of the study, under the supervision of the advisor, based on the literature on the subject and on the research question. A preliminary version of the instrument was assessed by a committee of specialists, who evaluated aspects of language comprehension, sequence of items and relevance of the questions to the purpose of the study. After the recommended adjustments, the instrument was composed of 54 closed questions, which explored: time since graduation, time working professionally, familiarity with online care, offering remote care during the pandemic, moving patients from face-to-face to remote mode, specific challenges of caring for adults and children and an open question, which asked the professional to briefly write about their experience of online care during the pandemic, highlighting the perceived challenges and advantages.

Participants confronted statements that had the following answer options: “I agree”, “I can’t say”, “I disagree”. For example: I feel more tired after performing remote sessions compared to face-to-face sessions; I had no difficulty adapting to the remote care; I’ve been facing some challenges to perform remote care; I feel fully prepared to assist remotely; I had questions about what was required under Federal Council of Psychology (FCP) guidelines to assist remotely; I believe that remote care is as effective as face-to-face care.

### Data analysis

Data were initially entered into Excel spreadsheets and then analyzed using the Statistical Package for Social Sciences - SPSS 28 ^( 23)^ . To avoid errors, data were entered by two members of the research team: the main researcher and a collaborating psychologist. After double typing, the versions were compared. Frequency counts were done manually using Excel, with subsequent comparison with SPSS results to ensure that there were no errors in transferring data from one program to another. 

The variables analyzed in the study were: sex, age, children, training time, experience with remote care, difficulties in adapting to the transition, facing challenges in this transition, feeling prepared for remote care, having doubts regarding the guidelines of the FCP, believing in the effectiveness of remote care. The variables that showed correlations were chosen to appear in the results of this study, considering a significance level of p ≤ 0.05. Cramér’s φ calculation was performed to verify the degree of association, taking into account the degree of freedom (df) of each analysis ^( 24- 26)^ . 

In addition, to test the hypothesis of association between qualitative variables, Pearson’s Chi-square *post-hoc* was performed, Adjusted Residuals (z) were calculated, to verify which of the categories contributed to a statistically significant result (p < 0.05). The absolute value of z was compared to the value 1.96, and the categories with absolute values of z greater than this, that is, that were placed outside the expected values for the null hypothesis were identified as those that contributed to a statistically significant result. Adjusted residuals less than -1.96 indicated that the category had a lower than expected value for the null hypothesis, and adjusted residuals greater than 1.96 indicated that the category had a greater than expected value for the null hypothesis ^( 24- 26)^ . The Cramér’s φ result was classified based on the literature consensus ^( 24- 25)^ . The degree of freedom was calculated for the X² test as = (number of rows -1) x (number of columns -1). 

### Ethical aspects

The study was approved by the Research Ethics Committee, under protocol n. 31863720 0 0000 5407 and the participants signed the Informed Consent Form online.

## Results

All regions of Brazil were represented in the sample, however, there was a higher concentration of participants from the Southeast region (n = 278, 72.2%), more specifically from the state of São Paulo (n = 238, 61.8%). Most of the sample consisted of women (n = 258), aged between 22 and 68 years old, mean of 35.41 years old (SD = 10.38) and range of 46 years old.

The main sociodemographic characteristics of the sample are systematized in Table 1. Regarding the results, in the question referring to the platforms used, psychologists could indicate more than one alternative, so that the total frequency could be greater than 385. 

 Sociodemographic characteristics of psychologists (n ^*^ = 385) with online assistance in the first year of the COVID-19 pandemic. Ribeirão Preto, SP, Brazil, 2020 

**Table 1 -:** Sociodemographic characteristics of psychologists (n * = 385) with online assistance in the first year of the COVID-19 pandemic. Ribeirão Preto, SP, Brazil, 2020

Sociodemographic Variables	f ^†^	% ^‡^
Sex		
Female	258	67.0
Male	127	33.0
Age group (years)		
22 a 30	154	40.0
30 a 40	139	36.1
> 40	92	23.9
Time since graduation (years)		
0 a 5	170	44.2
5 a 10	100	25.9
>10	115	29.9
Assisted online before the pandemic		
Yes	154	40.0
No	231	60.0
Platform(s) used for psychological care		
WhatsApp	277	71.9
Skype	185	48.0
Google Meet/Hangout	123	31.9
Zoom	55	12.3
Others	49	12.7

* n = Number of participants

† Corresponds to the number of answers in the category

‡ % = Percentage

### Difficulties encountered in the transition to remote care

When asked about how the transition from face-to-face to remote care was being experienced, more than half of the sample considered that online care was more tiring than face-to-face care (56.4%) and stated that they were facing some kind of challenge in this new modality (67.3%). A challenge pointed out by the participants was in relation to having doubts about the guidelines of the FCP (46.7%). Despite this, 51.9% of the sample stated that they felt fully prepared for this role and 54.3% did not consider that they were having difficulties in adapting to this transition. A fact that draws attention is that 47.7% of psychologists considered that remote care was not as effective as face-to-face care or did not yet feel qualified to give an opinion on this issue ( Table 2). 

**Table 2 -:** Distribution of frequencies and percentages of answers regarding remote care provided by psychologists in the first year of the COVID-19 pandemic (n * = 385). Ribeirão Preto, SP, Brazil, 2020

Affirmations	Answers						Total	
	Agree		I can’t say		Disagree			
	f ^†^	% ^‡^	f ^†^	% ^‡^	f ^†^	% ^‡^	f ^†^	% ^‡^
I feel more tired after performing remote care	217	56.4	68	17.7	100	26	385	100
I had no trouble adapting.	209	54.3	21	5.4	155	40.3	385	100
I have faced some challenges	259	67.3	42	20	84	21.8	385	100
I feel fully prepared	200	51.9	50	13	135	35.1	385	100
I had questions regarding the FCP Guidelines ^§^	180	46.7	57	14.8	148	38.4	385	100
Remote care is as effective as face-to-face care	205	53.2	47	12.2	133	35.5	385	100

* n =Number of participants

† Corresponds to the number of answers in the category

‡ % =Percentage

§ FCP = Federal Council of Psychology

### Variables related to difficulties in adapting to remote care

Among the variables analyzed, those that proved to be important, insofar as they were related to the presence of factors that hinder the process of adapting to remote psychological care in the first year of the COVID-19 pandemic, were: time since graduation and having previous experience with remote care.

Table 3 shows the cross tabulation between absence of difficulties in adapting to remote care and training time (p = .047). It was found that training time between five and 10 years was associated with greater perception of difficulties in adapting to remote care, however, the intensity of the association can be classified as small (Cramér’s φ = 0.112, df = 3). 

Table 4 shows the cross tabulation between previous experience with remote care before the COVID-19 pandemic and training time. Training time of less than five years was associated with having already performed remote care before the pandemic, however, the intensity of the association can be classified as small (Cramér’s φ = 0.150, df = 2). 

**Table 3 -:** Cross-tabulation between having no difficulty adapting to online care and training time for psychologists in remote care in the first year of the COVID-19 pandemic (n *=385). Ribeirão Preto, SP, Brazil, 2020

No difficulty adapting to remote care	Training time (years)					
	0 a 4	5 a 10	Over 10	Total	Cramér’s φ	p-value ^†^
Agree	95 (0.56)	44 (-2.40 ‡)	70 (1.69)	209	0.112	.047
I can’t say	12 (1.23)	7 (0.79)	2 (-2.10 ^‡^ )	21		
Disagree	63 (-1.14)	49 (2.07 ^‡^ )	43 (-0.75)	155		
Total	170	100	115	385		

* n = Number of participants

† p-value = Probability value, values in parentheses refer to Adjusted Residuals

**values with ^‡^ :**are absolute values greater than 1.96

**Table 4 -:** Cross tabulation between previous experience with remote care and training time (n *=385). Ribeirão Preto, SP, Brazil, 2020

Already performed remote care before the pandemic	Training time (years)					
	0 a 4	5 a 10	Over 10	Total	Cramér’s φ	p-valor ^†^
Yes	54 (-2.93 ^‡^ )	46 (1.42)	54 (1.82)	154	0.150	.013
No	116 (2.93 ^‡^ )	54 (-1.42)	61 (-1.82)	231		
Total	170	100	115	385		

* n = Number of participants

† p-value = Probability value, values in parentheses refer to Adjusted Residuals

**values with ^‡^ :**are absolute values greater than 1.96

It was found that the variable previous experience with remote care before the COVID-19 pandemic was the one that most showed associations, presenting itself as a facilitator of adaptation during the transition of modalities ( Table 5). In addition to training time, the following associations were found:

a) Perception of less difficulty in adapting to remote care (p =.004). The value of Cramér’s φ was 0.168, considering that the crossing has df = 2, the intensity of the relationship can be classified as small;
b) Less perception of having faced challenges in transposing face-to-face care to remote care (p = .022) and the association can be classified as small (Cramér’s φ = 0.141, df = 2);
c) Feeling fully prepared to assist in the remote format (p <.001), the association can be classified as moderate (Cramér’s φ = 0.343, df = 2);
d) Having no doubts about the FCP guidelines (p <.001), the association can be classified as moderate (Cramér’s φ = 0.221, df = 2).
e) Considering that remote care is as effective as face-to-face care and having already performed remote care before the COVID-19 pandemic (p = .002), the association can be classified as small (Cramér’s φ = 0.182, df = 2).

**Table 5-:** Cross tabulation between previous experience with remote care and other variables(n * = 385). Ribeirão Preto, SP, Brazil, 2020

Answers	Previous experience with remote care		Total	Cramér’s φ	p-value ^†^
	Yes	No			
No difficulty adapting to remote care					
Agree	99 (3.22 ^‡^ )	110 (-3.22 ^‡^ )	209	0.168	.004
I can’t say	8 (-0.18)	13 (0.18)	21		
Disagree	47 (-3.18 ^‡^ )	108 (3.18 ^‡^ )	155		
Total	154	231	385		
Faced challenges in moving to remote care					
Agree	92 (-2.57 ^‡^ )	167 (2.57 ^‡^ )	259	0.141	.022
I can’t say	18 (0.40)	24 (-0.40)	42		
Disagree	44 (2.62 ^‡^ )	40 (-2.62 ^‡^ )	84		
Total	154	231	385		
Feel fully prepared to assist remotely					
Agree	112 (6.66 ^†^ )	88 (-6.66 ^†^ )	200	0.343	<.001
I can’t say	14 (-1.86)	36 (1.86)	50		
Disagree	28 (-5.67 ^‡ ‡^ )	107 (5.67 ^‡ ‡^ )	135		
Total	154	231	385		
Questions regarding FCP guidelines ^§^					
Agree	54 (-3.75 ^‡^ )	126 (3.75 ^‡^ )	180	0.221	<.001
I can’t say	21 (-0.53)	36 (0.53)	57		
Disagree	79 (4.23 ^‡^ )	69 (-4.23 ^‡^ )	148		
Total	154	231	385		
Remote care as effective as face-to-face care					
Agree	98 (3.34 ^‡^ )	107 (-3.34 ^‡^ )	205	0.182	.002
I can’t say	11 (-2.48 ^‡^ )	36 (2.48 ^‡^ )	47		
Disagree	45 (-1.79)	88 (1.79)	133		
Total	154	231	385		

* Number of participants; the values in parentheses refer to the Adjusted Residuals

† p-value = Probability value

**values with ^‡^ :**are absolute values greater than 1.96

§ FCP = Federal Council of Psychology

## Discussion

This study aimed to offer an analysis of the difficulties encountered during the transition from face-to-face psychological care to the online mode, at the beginning of the COVID-19 pandemic in the Brazilian context, understanding that the availability of this format was crucial for the maintenance and recovery of health of the population. The sample consisted mostly of women, young professionals, with a predominance of activities in the private clinic. These data are compatible with the profile of the Brazilian psychologist^( 27)^. 

Compulsory isolation produced changes that impacted social interaction and led to changes in the way of living and dying that were accompanied by intense psychological suffering, increasing the demand for psychological care^( 19)^. In this scenario, professionals needed to adjust to the changes introduced in the therapeutic setting, which required flexibility from the psychotherapist and new learning in a short period of time^( 28)^. One of the risks of lack of adequate training may be the tendency to transpose the face-to-face model to the remote one, ignoring the particularities of each one, with greater difficulty in carrying out a critical, ethical and technical analysis to determine the best possibility of service for each presented demand^( 16)^. 

The feeling that remote care was more tiring than face-to-face care stands out, which must be understood in the broader context of the pandemic, in which professionals were also subjected to the restrictive and threatening conditions that characterized the pandemic moment. In addition to providing relief for the emotional suffering of their patients, psychologists often had to deal with their own vulnerability and that of their families. Another data to be considered is the very specificity of care mediated by technology, which required time in front of the screen, greater need for concentration and the fact that they had to deal with work demands and family care, which were superimposed on the same environment due to domestic confinement^( 29)^. 

When analyzing the factors that facilitated the transition to the remote mode of care, the following stand out: time since graduation and previous experience with this type of intervention. It was found that psychologists with less than five years in the profession had less difficulty in adapting, which may be linked to the generational factor, with greater prior contact with the digital environment. The lack of familiarity with the management of Information and Communication Technologies may have hindered the sudden and emergency change to the virtual setting, which occurred without the necessary training^( 30)^ that could have helped in adjusting to the technical and ethical issues introduced in the therapeutic setting. 

The subgroup with less than five years of training concentrated a greater number of professionals with previous experience in the remote mode of care before the pandemic. Previous experience was the variable that stood out the most in the results, being associated with a lower perception of difficulties in adapting to remote care, the experience of minor challenges in transposing face-to-face to online care, feeling fully prepared for this format, not having doubts about the guidelines of the FCP and considering remote care as effective as face-to-face care.

The lack of experience in remote care prior to the pandemic seems to be related to distrust regarding the effectiveness of the modality mediated by technology resources. However, this distrust may also be due to the non-inclusion of this content in the professional training of psychologists, since most respondents stated that they had not had contact with this type of service during their undergraduate internships. This fact appears to be related to the experience of greater challenges in adjusting to changes, the perception of not being qualified for remote care and lack of knowledge of the guidelines of the Federal Council of Psychology. This lack of knowledge was evident in the observation that the answers to some of the questions mentioned by the survey respondents could be found in Resolutions made available to professionals. This data suggests that they were not having access to the materials produced or that the dissemination of guidelines was not having a satisfactory reach.

The effectiveness of remote therapy was questioned by psychologists even before the pandemic, despite studies finding similar effects with the application of both modalities, and in the pandemic context, due to the need for almost exclusive use of this type of intervention, these questions acquired an even greater weight^( 31)^. It was evident that, for most psychologists, offering technology-mediated psychological interventions was fundamental at the pandemic, when multiple challenges were superimposed. The online format showed real potential for emotional help for the patients treated, functioning as a means of protection, prevention and promotion of mental health. 

The growing tendency to transfer life to the virtual universe and concerns about the impact of technology on everyday relationships are open questions that still require investments in a consistent research agenda. Considering this complex scenario, it is worth mentioning that there are special considerations for professional practices in the remote format. However, the questions and concerns expressed by professionals were often at an intrapersonal level, starting with the compulsory break with the classic models of psychotherapeutic practice. On the other hand, aspects of the organization of the profession in the country and the few advances obtained in the issue of the use of Information and Communication Technologies compared to the exponential increase in the population’s access to different technologies in recent years are also challenges to be overcome by the category of psychologists.

More than just looking for immediate practical answers, these questions should encourage reflection and drive conceptual expansions and transformations in professional practice. Therefore, it is considered, based on the results obtained, that the reverberations produced by the pandemic provoked the need for updates and reconfigurations of professional practice that did not happen in a single or immediate way. The repositioning of practices and knowledge is in process^( 32)^ and undergraduate courses must consider this new reality in order to also resize their training proposals in view of the new needs. 

A limitation of this study is the limit of the methodological strategy used: the answers were based on self-report and it is not possible to have effective control over who was actually accessing the platform and answering to the form. Another aspect that can be highlighted is that, despite including a sample of clinical psychologists with representatives from all regions of the country, the highest concentration of respondents is located in the Southeast region, more specifically in the state of Sao Paulo, which implies limits to the generalization of the results on the transition experience from the face-to-face mode to remote care. It is suggested that further research be carried out in other Brazilian scenarios, aiming to verify how the transition to the remote modality was experienced, with its difficulties and facilities. On the other hand, the fact that the data were collected in the “heat of the moment”, that is, in full force of a critical phase of the pandemic, can be pointed out as a strength of the study.

Another important contribution of the study is the finding that previous experience with remote care stood out as a facilitating factor for the transition from psychological care, which was previously carried out in person. This suggests the need to insert, in the curriculum of undergraduate courses in Psychology (and possibly in other courses in the health area) contents related to the use of technologies as mediators of care, less from the instrumental point of view and more from the perspective of critical thinking, guided by ethical principles and dictates regarding the limits and potentialities of the use of digital resources.

Health professionals were challenged to learn how to care and teach care remotely. Digital tools that were not used before became central, especially in the hospital, enabling tele follow-up, telemonitoring, virtual visits, teleguidance, tele inter consultations and team meetings^( 8)^. Professional areas of health, such as nursing and psychology, usually value (and often need) physical contact to deepen the bond with the patient, whether to implement the intervention or for theoretical-practical teaching. Thus, professors need to be prepared to handle this tool in new teaching modalities^( 22)^. 

## Conclusion

The following were identified as facilitating factors for adapting to the change from the face-to-face to the online setting: shorter training time and previous experience with remote care. The biggest obstacles were: having between five and 10 years of graduation and not having previous familiarity with the use of technologies.

The variable that most stood out as a facilitator in the transition from face-to-face to remote care was previous experience with remote care. This suggests the need to include content on the limits and possibilities of using technologies as mediating resources in psychological care in the curriculum of undergraduate courses.
